# Stimulating the Addictive Brain

**DOI:** 10.3389/fnhum.2012.00220

**Published:** 2012-07-30

**Authors:** Jens Kuhn, Theo O.J. Gruendler, Joachim Klosterkötter, Christina Bartsch

**Affiliations:** ^1^Department of Psychiatry and Psychotherapy, University of CologneCologne, Germany; ^2^Max Planck Institute for Neurological ResearchCologne, Germany

In western industrialized nations approximately 25% of all deaths are caused directly or indirectly by the consumption of psychotropic substances. Substance-related addictions therefore constitute the most frequently occurring psychiatric disease category (McGinnis and Foege, 1993). The etiology of addiction due to psychoactive substances is highly multi-factorial. These factors being social and environmental influences, a person's individual biography, personality traits, and genetic predisposition as far as the susceptibility to substance-dependence is concerned. However, from a neuropathophysiological point of view, addiction is a chronically recurring disease of the brain resulting from a dysregulation of neural circuits. Especially a dysregulation of the mesocorticolimbic pathway, including the nucleus accumbens (NAcc), is considered to play a major role in this context (Adinoff, 2004). Recent understanding of the neural pathways affected in addiction has created a new range of treatment options that directly target dysregulated brain circuits in order to normalize functionality, e.g., deep brain stimulation (Luigjes et al., 2012). However, what remained unknown so far were the consequences of psychoactive drug abuse taking place on a cellular level and eventually leading toward dysfunctional circuitry.

In their recently published manuscript Pascoli et al. (2011) elucidate the molecular mechanisms following cocaine exposure. By using an animal model the authors demonstrate that cocaine potentiates the dopaminergic transmission in D1 expressing accumbal neurons. These early changes in synaptic plasticity may initiate cocaine dependency and thereby precede the pathological changes that characterize addictive behavior. Most interestingly, these cocaine evoked neuroplastic changes are reversed under an inhibitory optogenetic stimulation procedure. The impact of these findings for new experimental therapies, such as transcranial magnetic stimulation and DBS, is touched on by the authors but is – according to our judgment – even of greater relevance than it is expressed in the article.

Since the first description of a patient, whose alcohol addictive behavior was positively influenced by high-frequency accumbal DBS (Kuhn et al., 2007), additional case reports hint to a beneficial therapeutic effect of the method in addiction (for an overview, see Luigjes et al., 2012). By mimicking different aspects of addiction, translational animal research indicates that stimulation of the nucleus accumbens has a significant positive impact on related behavioral patterns (Liu et al., 2008; Vassoler et al., 2008; Knapp et al., 2009; Henderson et al., 2010). The generalizability of these beneficial effects of DBS on different drug-addictions has yet to be established but seems promising given the broad range of psychotrophic substances, including cocaine, alcohol, or opioids, tested in animal models.

Although the mechanisms of deep brain stimulation are, despite its decade-long application, not yet fully understood it has been argued that the modulation of dopaminergic transmission – especially in the context of addictive disorders – underlies the therapeutic effect. While this assumption could only be demonstrated for the rat nucleus accumbens (Sesia et al., 2010), PET-imaging studies in humans show alterations of dopamine release and dopamine binding potential under thalamic stimulation (Kuhn et al., 2012). The regulative function of the ventral striatum, including the nucleus accumbens, however, is not only relevant to addiction but also to other psychiatric disorders. Its unimpaired functioning, however, does not only depend on a subtle interaction between D1 and D2 receptor activations (Goto and Grace, 2008) but also on a D1-associated co-activation of NMDA-receptors, thereby also involving the glutamatergic system (Lee et al., 2011). Furthermore, the differential involvement of receptor types and the interaction of tonic levels and phasic burst and the timing of synaptic availability of dopamine add to the complexity of these regulatory processes.

The influence of impaired dopaminergic functioning on cognition and neuronal plasticity has been much debated. It is assumed that an activation of accumbal D1 receptors by phasic dopamine release facilitates limbic afferents and promotes cortical synaptic plasticity. At the same time, D2 receptor activation might promote the reinforcement of prefrontal influences, which is especially relevant in the context of addiction given the reduced density of both striatal and accumbal D2 receptors in addicted patients (Lee et al., 2011; Volkow et al., 2011).

The results of Pascoli et al. (2011) help explain the concept of an impaired nucleus accumbens function in addiction (Adinoff, 2004) by pointing to the selective impact of psychotrophic substances on the neuroplasticity of D1 receptor neurons and a possible restoration through focal long-term depression.

The short-term application of psychotropic substances, in this case cocaine, apparently triggers a fast modification of D1-receptor expression in terms of focal synaptic plasticity and could, hence, bring forth a lasting shift in the D1/D2 ratio, which eventually might lead to a fortification of limbic and drug-associated afferents.

Current pharmacologic approaches to ameliorate the dysregulated dopaminergic system are not sufficiently effective (Spanagel and Vengeline, 2012). Yet, selective inhibitory optogenetic stimulation is able to reverse early drug associated alterations and thus potentially interrupts the cascade leading to or upholding addiction (Pascoli et al., 2011). In our opinion, this new model of NAcc interaction and manipulation through stimulation supports the application of DBS as a promising approach in the treatment of otherwise refractory severe substance addiction.
